# Stroke risk stratifications according to CHA_2_DS_2_-VASc vs. CHA_2_DS_2_-VA in patients with atrial fibrillation: insights from the GLORIA-AF registry

**DOI:** 10.1093/ehjcvp/pvaf031

**Published:** 2025-04-28

**Authors:** Steven Ho Man Lam, Giulio Francesco Romiti, Bernadette Corica, Tommaso Bucci, Brian Olshansky, Tze-Fan Chao, Menno V Huisman, Gregory Y H Lip

**Affiliations:** Liverpool Centre for Cardiovascular Sciences at University of Liverpool, Liverpool John Moors University and Liverpool Heart & Chest Hospital, Liverpool L7 8TX, UK; Department of Medicine and Therapeutics, The Chinese University of Hong Kong, Hong Kong, China; Liverpool Centre for Cardiovascular Sciences at University of Liverpool, Liverpool John Moors University and Liverpool Heart & Chest Hospital, Liverpool L7 8TX, UK; Department of Translational and Precision Medicine, Sapienza—University of Rome, Rome, Italy; Liverpool Centre for Cardiovascular Sciences at University of Liverpool, Liverpool John Moors University and Liverpool Heart & Chest Hospital, Liverpool L7 8TX, UK; Liverpool Centre for Cardiovascular Sciences at University of Liverpool, Liverpool John Moors University and Liverpool Heart & Chest Hospital, Liverpool L7 8TX, UK; Department of Clinical Internal, Anesthesiologic and Cardiovascular Sciences, Sapienza University of Rome, Rome, Italy; Division of Cardiology, Department of Medicine, University of Iowa, 200 Hawkins Drive, Iowa City, IA 52242, USA; Division of Cardiology, Department of Medicine, Taipei Veterans General Hospital, No. 201, Sec. 2, Shipai Rd., Beitou District, Taipei 11217, Taiwan; Institute of Clinical Medicine, and Cardiovascular Research Center, National Yang Ming Chiao Tung University, No. 155, Sec. 2, Linong St., Beitou Dist., Taipei 112304, Taiwan; Department of Thrombosis and Hemostasis, Leiden University Medical Center, Leiden, The Netherlands; Liverpool Centre for Cardiovascular Sciences at University of Liverpool, Liverpool John Moors University and Liverpool Heart & Chest Hospital, Liverpool L7 8TX, UK; Department of Clinical Medicine, Danish Centre for Health Services Research, Aalborg University, Aalborg, Denmark; Medical University of Bialystok, Bialystok, Poland

**Keywords:** Atrial fibrillation, Stroke, CHA_2_DS_2_-VASc, CHA_2_DS_2_-VA

## Abstract

**Aims:**

Whether the adoption of CHA_2_DS_2_-VA score, the sex-independent version of the CHA_2_DS_2_-VASc score is beneficial for stratifying risk of stroke in patients with atrial fibrillation (AF) remains controversial.

**Methods and results:**

Utilizing the data from the global, multicentre and prospective GLORIA-AF Registry Phase III, we compared the performances of CHA_2_DS_2_-VA and CHA_2_DS_2_-VASc scores in stratifying the risk of ischaemic stroke and thromboembolism (TE), and compared the risk of ischaemic stroke and TE, and the use of oral anticoagulants in male and female patients with AF. A total of 21 260 AF patients with available data were included in the analysis (mean age 70.2 ± 10.3 years, 44.9% female). Overall, female patients were less likely prescribed with oral anticoagulant (OAC) compared with males [odds ratio: 0.90, 95%confidence interval [CI]: (0.83–0.97)]. A significant interaction (*P* < 0.001) between sex and age was observed, with a lower likelihood of receiving OAC among younger female patients.

The risk of ischaemic stroke [hazard ratio (HR):1.14, 95%CI: (0.85–1.53)] and TE [HR: 1.02, 95%CI: (0.82–1.26)] was similar between male and female patients, and the predictive ability of the two scores was similar for both outcomes: TE [area under the receiver operating characteristic curve (AUC): 0.641, 95%CI: (0.585–0.697) vs. AUC: 0.636, 95%CI: (0.580–0.692); *P* = 0.593] and ischaemic stroke [AUC: 0.660, (95%CI: 0.582–0.739) vs. AUC: 0.646, (95%CI: 0.568–0.725); *P* = 0.847]. There was a possible interaction between sex and age observed, with a higher risk of TE in younger female patients (*P* = 0.051).

**Conclusion:**

CHA2DS2-VA score had similar predictive performance for thromboembolic events compared with CHA_2_DS_2_-VASc score. The role of female sex in the management and outcomes of patients with AF may differ according to age. Whether the omission of the female criterion from CHA_2_DS_2_-VA would lead to less OAC use in female AF patients over the next years remains to be seen.

## Introduction

Atrial fibrillation (AF) remains the most common arrhythmia, contributing to an increased risk of stroke, heart failure, dementia, and cardiovascular death.^1–3^ Historical data from up to two decades ago indicated a higher burden of stroke risk in female patients with AF than in male patients;^4,5^ moreover, compared with males, female patients with AF tended to be undertreated with oral anticoagulants (OACs), although this disparity has declined in recent years.^6,7^ Given the evidence from older studies, female sex was incorporated as a risk factor for ischaemic stroke in patients with AF.^8^ With more recent evidence being available, the risk associated with female sex is suggested to be age-dependent, and other authors advocated for its role as a ‘risk modifier’ rather than a risk factor *per se*.^9^

Contemporary data have shown an overall decline in ischaemic stroke rates associated with AF, and the difference in stroke rates between female and males has become less significant in recent years.^10,11^ Whether the narrowing gap in the ischaemic strokes rates between women and men should lead to removing the sex criterion from the CHA_2_DS_2_-VASc score remains an open question. The 2024 European Society of Cardiology guidelines has advocated for the use of CHA_2_DS_2_-VA score (with Level of Evidence C) ‘in the absence of other locally validated alternatives’, since ‘the inclusion of gender complicates clinical practice … (and) omits individuals who identify as non-binary, transgender, or are undergoing sex hormone therapy.’^12^

Nevertheless, recent well-conducted large observational studies have shown similar predictive ability of CHA_2_DS_2_-VASc and CHA_2_DS_2_-VA (i.e. without considering sex) scores.^13,14^ Another UK study examining the impact of female sex excluded AF patients aged ≥75 years and those with prior stroke, thus comparing CHD-VASc and CHD-VA scores, and not CHA_2_DS_2_-VASc and CHA_2_DS_2_-VA *per se*.^15^ Nonetheless, uncertainties on the adoption of CHA_2_DS_2_-VA, and the removal of sex criterion from the stroke risk assessment model have been raised.^16^

The aim of this analysis is to formally compare CHA_2_DS_2_-VASc and CHA_2_DS_2_-VA in terms of thromboembolic risk stratification, in a contemporary and global prospective cohort of patients with AF.

## Methods

### Study design

To perform this analysis, we used data from the Phase III of the Global Registry on Long-Term Oral Antithrombotic Treatment in Patients with Atrial Fibrillation (GLORIA-AF) Registry. Full details of the protocol, study design, methodology, and primary results of the GLORIA-AF Registry had been published before.^17,18^ In brief, GLORIA-AF is a global, multicentred, prospective registry programme composed of three phases, with the aim of evaluating the safety and effectiveness of dabigatran etexilate in patients with a recent diagnosis of AF (<3 months or <4.5 in Latin America). Detailed inclusion and exclusion criteria had been previously described;^17^ briefly, to be eligible for inclusion, patients must be 18 years or older, with a recent diagnosis of non-valvular AF and a CHA_2_DS_2_-VASc score ≥1. Phase III of the registry was performed between 2014 and 2016, and patients were enrolled and followed for 3 years regardless of their antithrombotic treatment received at baseline. Ethics approvals were received from local institutional review boards, and all patients provided their written informed consents. The study was carried out in accordance with the Declaration of Helsinki.

### Data collection

Demographics, clinical, and treatment data were collected with standardized electronic case report form across all sites worldwide. At baseline, investigator recorded all the criterion of the risk scores, including age, sex, and history of heart failure, hypertension, stroke, vascular disease, and diabetes. CHA_2_DS_2_-VASc score was calculated at baseline; for the purpose of this analysis, CHA_2_DS_2_-VA was calculated by subtracting 1 to CHA_2_DS_2_-VASc in female patients, and as CHA_2_DS_2_-VASc in males. Antithrombotic treatment at baseline was also recorded with the following details: whether the patient received oral anticoagulant (OAC) and the type of OAC [either vitamin-K antagonist (VKA) or non-VKA oral anticoagulant (NOAC)], antiplatelets or no antithrombotic treatment.

### Study outcomes

Our primary outcome was thromboembolism [TE, defined as the composite of stroke, transient ischaemic attack (TIA), and other non-central nervous system TE] at 1 year after baseline; patients were therefore censored at 365 days of follow-up for the purpose of this analysis. We additionally evaluated ischaemic stroke as individual outcome. The incidence rates (IR) were compared between different risk levels classified by the two risk scores (CHA_2_DS_2_-VASc vs. CHA_2_DS_2_-VA), and across sexes.

### Statistical analysis

Continuous variables and categorical variables were reported either as mean and standard deviation (SD) for normally distributed data or as median and inter-quartile range (IQR) for non-normally distributed data, and count with percentage, respectively. Baseline characteristics between sexes were compared with χ^2^ test, Student’s *t*-test, or Mann–Whitney U-test for categorical variables, normally distributed continuous variables and non-normally distributed continuous variables, respectively. Incidence rates were calculated, per 100 patient-years, using the epiR package; corresponding 95%confidence interval (CI) were calculated with exact method. Incidence rate ratio (IRR) for females vs. males was reported, and corresponding 95%CI were calculated using the fsmb package.

Receiver operating characteristic (ROC) curves were drawn to illustrate the predictive performances of the two risk scores and the area under the ROC curves (AUCs) were used to quantify the overall predictive abilities of the two risk scores. De Long test was used to statistically compare the difference in AUCs between the risk scores. Additionally, integrated discrimination improvement index (IDI) and continuous net reclassification index (NRI) were calculated at 1 year. We additionally explored the association of female sex with use of OAC at baseline using multiple logistic regression models, which were adjusted for the other components of the CHA_2_DS_2_-VA (age category, hypertension, diabetes, heart failure, coronary artery disease, peripheral artery disease, and history of previous stroke/TIA), and the interaction of female sex with age (modelled as a restricted cubic spline with three knots) on the odds of receiving OAC at baseline, after adjustment for the other components of the CHA_2_DS_2_-VA, beyond age. Finally, we also explored the association of female sex with the risk of ischaemic stroke and TE at 1 year, using multiple Cox regression models, adjusted for the other components of the CHA_2_DS_2_-VA and the use of OAC at baseline; the interaction of female sex with age (modelled as a restricted cubic spline with three knots) on the risk of outcomes was also assessed, after adjustment for the other components of the CHA_2_DS_2_-VA (beyond age) and use of OAC. All statistical analyses were performed with R version 4.3.1 (R Core Team 2020, Vienna, Austria), and a two-sided *P* < 0.05 was considered statistically significant.

## Results

### Baseline characteristics

A total of 21 260 patients with available data were included in the analysis (mean age 70.2 ± 10.3 years, 44.9% female). *Table 1* presents the baseline characteristics of the cohort. At baseline, female patients were older, were more likely to have lower body mass index (BMI) and lower systolic blood pressure. They also had a lower prevalence of heart failure, coronary artery disease, peripheral artery disease, diabetes, previous bleeding, abnormal kidney function, and chronic obstructive pulmonary disease (*P* < 0.001 for all).

**Table 1:** Baseline characteristics according to sex

Variables	Males (*n* = 11 717)	Females (*n* = 9543)	*P*
Age, mean (SD)	68.8 (10.5)	71.9 (9.8)	<0.001
BMI, median [IQR]	27.6 [24.7–31.2]	27.3 [24.0–31.6]	<0.001
Region, *n* (%)			<0.001
North America	2798/11 717 (23.9)	2316/9543 (24.3)	–
Europe	5627/11 717 (48.0)	4661/9543 (48.8)	–
Asia	2442/11 717 (20.8)	1791/9543 (18.8)	–
Other	850/11 717 (7.3)	775/9543 (8.1)	–
AF type and symptoms, *n* (%)			
Paroxysmal AF	6229/11 717 (53.2)	5751/9543 (60.3)	<0.001
Persistent AF	4341/11 717 (37.0)	2920/9543 (30.6)	–
Permanent AF	1147/11 717 (9.8)	872/9543 (9.1)	–
EHRA III–IV	3246/11 717 (27.7)	3359/9543 (35.2)	<0.001
Medical history, *n* (%)			
Hypertension	8775/11 692 (75.1)	7081/9521 (74.4)	0.264
Heart failure	2833/11 625 (24.4)	1791/9460 (18.9)	<0.001
CAD	2689/11 420 (23.5)	1305/9315 (14.0)	<0.001
Diabetes mellitus	2931/11 717 (25.0)	2020/9543 (21.2)	<0.001
PAD	425/11 599 (3.7)	196/9505 (2.1)	<0.001
Previous stroke/TIA	1733/11 716 (14.8)	1304/9543 (13.7)	0.021
Previous bleeding	680/11 480 (5.9)	450/9379 (4.8)	<0.001
Abnormal kidney function	265/11 562 (2.3)	126/9433 (1.3)	<0.001
COPD	763/11 567 (6.6)	521/9465 (5.5)	0.001
Dementia	54/11 568 (0.5)	70/9458 (0.7)	0.013
History of cancer	1179/11 536 (10.2)	944/9420 (10.0)	0.651
Antithrombotic treatment, *n* (%)			0.041
None	725/11 712 (6.2)	658/9537 (6.9)	–
Antiplatelets	1349/11 712 (11.5)	1015/9537 (10.6)	–
NOAC	6957/11 712 (59.4)	5717/9537 (59.9)	–
VKA	2681/11 712 (22.9)	2147/9537 (22.5)	–
CHA2DS2-VASc, *n* (%)			
1	2576/11 717 (22.0)	493/9543 (5.2)	–
2	3145/11 717 (26.8)	1388/9543 (14.5)	–
3	2915/11 717 (24.9)	2444/9543 (25.6)	–
4	1703/11 717 (14.5)	2594/9543 (27.2)	–
5	923/11 717 (7.9)	1490/9543 (15.6)	–
6	345/11 717 (2.9)	800/9543 (8.4)	–
7	99/11 717 (0.8)	267/9543 (2.8)	–
8	11/11 717 (0.1)	56/9543 (0.6)	–
9	0/11 717 (0.0)	11/9543 (0.1)	–
CHA2DS2-VA, *n* (%)			
0	0/11 717 (0.0)	493/9543 (5.2)	–
1	2576/11 717 (22.0)	1388/9543 (14.5)	–
2	3145/11 717 (26.8)	2444/9543 (25.6)	–
3	2915/11 717 (24.9)	2594/9543 (27.2)	–
4	1703/11 717 (14.5)	1490/9543 (15.6)	–
5	923/11 717 (7.9)	800/9543 (8.4)	–
6	345/11 717 (2.9)	267/9543 (2.8)	–
7	99/11 717 (0.8)	56/9543 (0.6)	–
8	11/11 717 (0.1)	11/9543 (0.1)	–

BMI, body mass index; CAD, coronary artery disease; COPD, chronic obstructive pulmonary disease; EHRA, European Heart Rhythm Association; IQR, inter-quartile range; PAD, peripheral artery disease; SD, standard deviation; TIA, transient ischaemic attack.

### Use of antithrombotic treatment at baseline

**Figure 1:**
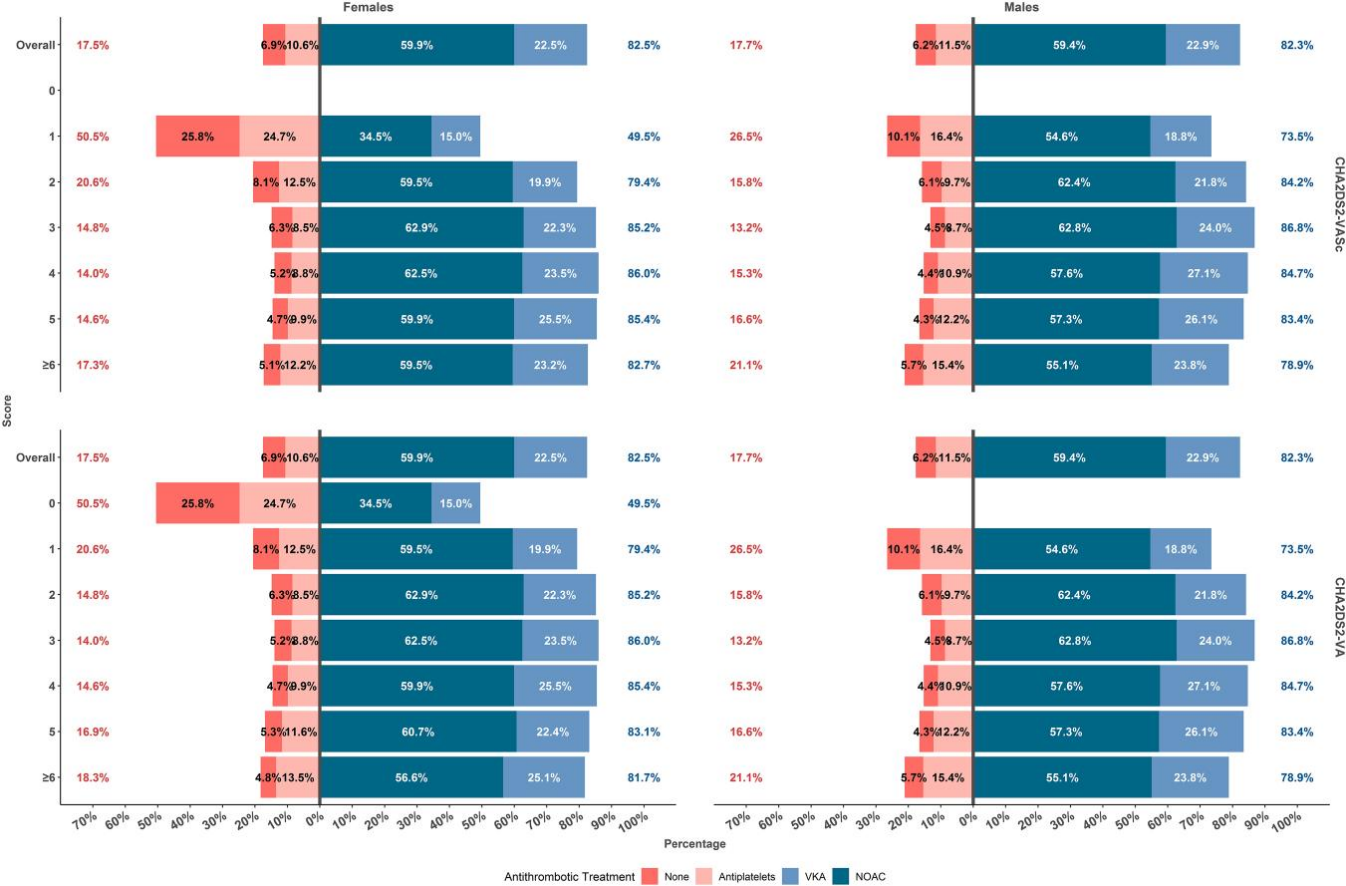
Antithrombotic treatment according to sexes and scores. NOAC, non-vitamin-K antagonist oral anticoagulant; VKA, vitamin-K antagonist.

Distribution of antithrombotic treatment at baseline according to the two scores is reported in Supplementary material online, *Figure S1* (patients with scores ≥6 were considered together), while distribution across sexes and by scores is reported in *Figure 1*. Overall, 82.4% of the patients received OAC (59.6% NOAC, 22.7% VKA); 6.5% did not receive any antithrombotic treatment, while 11.1% were treated with antiplatelets. Use of OAC was numerically similar in females (82.5%; 59.9% received NOAC) and males (82.3%; 59.4% received NOAC; *Figure 1*). The uptake of OAC increased as the CHA_2_DS_2_-VASc and CHA_2_DS_2_-VA increased, particularly in females; among both sexes, use of OAC was slightly lower in those with scores ≥6 (*Figure 1*).

**Figure 2:**
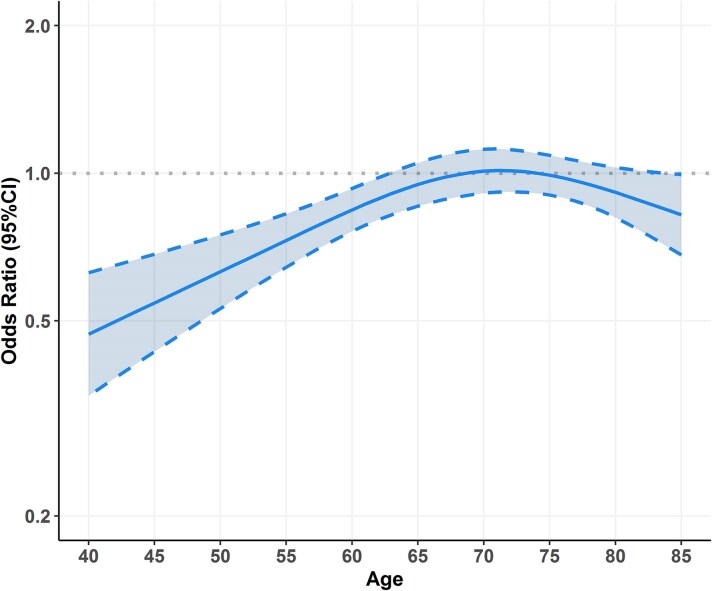
Odds of receiving oral anticoagulant in females vs. males according to age. CI, confidence interval.

When we analysed the odds of receiving OAC according to sex, and after adjustments for other factors of the CHA_2_DS_2_-VA score, we found that females were less likely prescribed with OAC compared with males [odds ratio (OR): 0.90, 95%CI: 0.83–0.97]; in the exploratory analysis of the interaction of sex with age (modelled as a restricted cubic spline) on the odds of receiving OAC, we observed a significant interaction (*P* < 0.001) of age with likelihood of receiving OAC in females, with younger females patients less likely to receive OAC at baseline (*Figure 2*).

### Incidence rates of thromboembolism and ischaemic stroke

**Figure 3:**
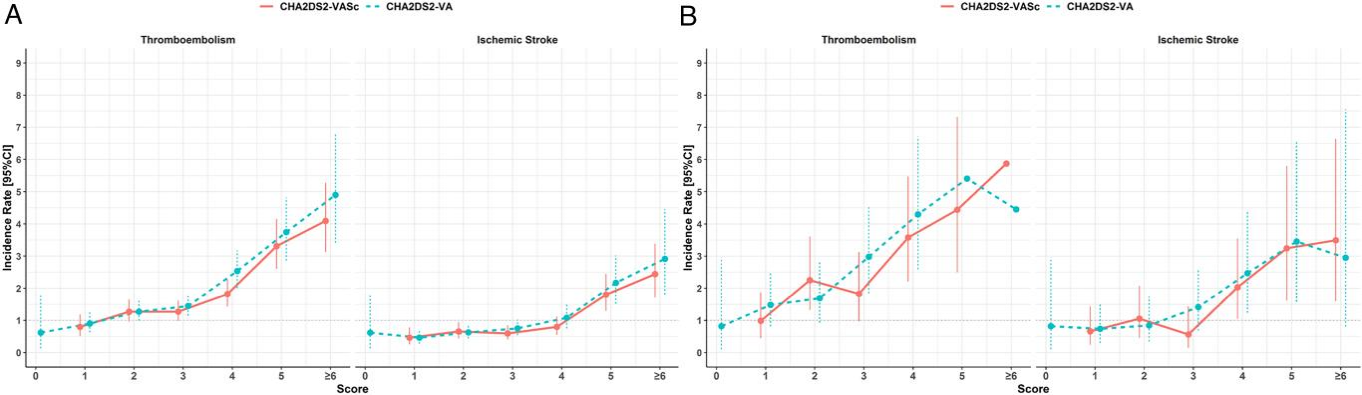
(*A*) Incidence rates and 95% confidence intervals of thromboembolism and ischaemic stroke, according to scores in the whole cohort (left); (*B*) incidence rates and 95%confidence intervals of thromboembolism and ischaemic stroke, according to scores, in non-anticoagulated patients (right). CI, confidence intervals.

Incidence rates and corresponding 95%CI at 1 year for TE and ischaemic stroke, stratified by scores are reported in *Figure 3A* and Supplementary material online, *Table S1*, while IR and 95%CI stratified by sex and scores are reported in Supplementary material online, *Figure S2*. Incidence rates of TE were slightly higher for each level of CHA_2_DS_2_-VA compared with CHA_2_DS_2_-VASc; similar results were observed for ischaemic stroke when scores were ≥3 (*Figure 3A*). When stratifying by sex, males showed broadly higher IR of TE compared with females for each stratum of the CHA_2_DS_2_-VASc; conversely, similar rates were observed for ischaemic stroke, and when analysing IR of both events according to CHA_2_DS_2_-VA scores (see Supplementary material online, *Figure S2*). No statistically significant differences were observed for IRR in females vs. males, across each stratum of the two scores (see Supplementary material online, *Table S1*).

### Risk of thromboembolism and ischemic stroke

**Figure 4:**
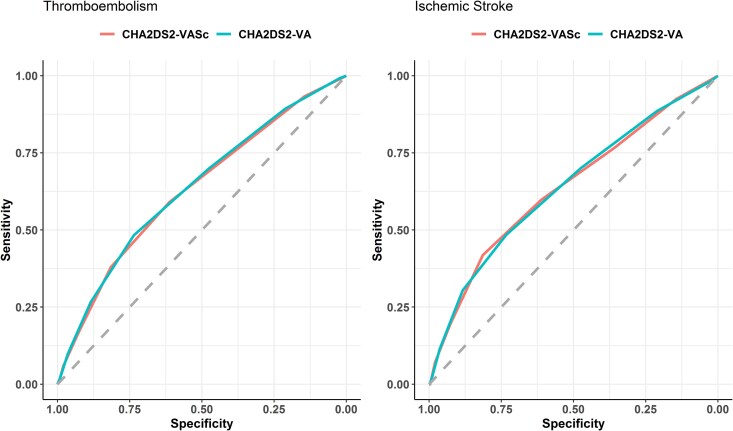
Receiver operating characteristic curves for thromboembolism and ischaemic stroke, according to scores.

Receiver operating characteristic curves for CHA_2_DS_2_-VASc and CHA_2_DS_2_-VA, for TE and ischaemic stroke, are reported in *Figure 4*. No significant differences were observed in terms of predictive performance for both TE [AUC: 0.632 (95%CI: 0.602–0.661) and 0.637 (0.607–0.666) for CHA_2_DS_2_-VASc and CHA_2_DS_2_-VA, respectively; *P* = 0.294] and for ischaemic stroke [AUC: 0.638 (95%CI: 0.595–0.680) and 0.639 (0.597–0.681) for CHA_2_DS_2_-VASc and CHA_2_DS_2_-VA, respectively; *P* = 0.856]. No statistically significant differences were observed when analysing IDI and NRI for CHA_2_DS_2_-VA vs. CHA_2_DS_2_-VAsc (see Supplementary material online, *Table S3*).

When we analysed the risk of ischaemic stroke and TE according to sex, and after adjustment for the other components of the CHA_2_DS_2_-VA score and the use of OAC, we did not find a higher risk of ischaemic stroke or TE at 1 year in females [hazard ratio (HR): 1.14, 95%CI: 0.85–1.53 and HR: 1.02, 95%CI: 0.82–1.26, respectively].

We then explored the interaction of sex and age (modelled as a restricted cubic spline) on the risk of outcomes; there was evidence of a marginally significant interaction for TE (*P* = 0.051), with risk that tended to increase in younger females compared with younger male patients (see Supplementary material online, *Figure S3*, left panel). We did not observe any interaction for ischaemic stroke (*P* = 0.349; Supplementary material online, *Figure S3*, right panel).

### Sensitivity analysis in non-anticoagulated patients

We performed a sensitivity analysis restricted to patients who did not receive anticoagulation at baseline [*n* = 3747; 1667 (44.5%) females]. Of these, 2815 (75.1%) had a CHA_2_DS_2_-VASc ≥2 [1391 (67.1%) among males, 1424 (85.1%) among females]; there were 249 females with a CHA_2_DS_2_-VA score of 0, while 932 (24.9%) patients had a CHA_2_DS_2_-VASc of 1 (683 males and 249 females).

Incidence rates and 95%CI according to the two scores for TE and ischaemic stroke and stratified by sex are reported in Supplementary material online, *Table S2* and in *Figure 3B*. For both TE and ischaemic stroke, IR were similar between males and females when using CHA_2_DS_2_-VASc; conversely, females showed slightly higher rates of both TE and ischaemic stroke across strata of CHA_2_DS_2_-VA, particularly for score ≥3.

When we compared ROC curves, we found similar predictive ability of the two scores for both TE [AUC: 0.641 (95%CI: 0.585–0.697) and 0.636 (0.580–0.692) for CHA_2_DS_2_-VASc and CHA_2_DS_2_-VA, respectively; *P* = 0.593] and ischaemic stroke [AUC: 0.660 (95%CI: 0.582–0.739) and 0.646 (0.568–0.725) for CHA_2_DS_2_-VASc and CHA_2_DS_2_-VA, respectively; *P* = 0.847] (see Supplementary material online, *Figure S4*). No statistically significant differences were observed when analysing IDI and NRI for CHA_2_DS_2_-VA vs. CHA_2_DS_2_-VAsc (see Supplementary material online, *Table S4*).

We finally analysed the risk of TE and ischaemic stroke according to sex; after adjustment for the other components of the CHA_2_DS_2_-VA score, we found that female patients had the higher risk of ischaemic stroke at 1 year (HR: 1.86, 95%CI: 1.03–3.33); similar results, albeit non-statistically significant, were observed for TE (HR: 1.48, 95%CI: 0.97–2.27). When we explored the interaction of sex and age (modelled as a restricted cubic spline) on the risk of outcomes, we did not observe any significant interaction for TE (*P* = 0.312; Supplementary material online, *Figure S5*, left panel) and ischaemic stroke (*P* = 0.577) (see Supplementary material online, *Figure S5*; right panel).

## Discussion

In this exploratory analysis of the CHA_2_DS_2_-VASc and CHA_2_DS_2_-VA scores in a contemporary global, prospective cohort of AF patients, our main findings are as follows: (i) the performances of CHA_2_DS_2_-VA and CHA_2_DS_2_-VASc scores for predicting TE and ischaemic stroke were similar; (ii) female patients were less likely to receive OAC at baseline, when adjusting for the other factors of the CHA_2_DS_2_-VA score; and (iii) the association of female sex with odds of receiving OAC and risk of thromboembolic outcomes differed according to age.

The results indicate that both CHA_2_DS_2_-VA (AUC: 0.637) and CHA_2_DS_2_-VASc (AUC: 0.632) modestly predicted TE incidence, with both AUCs <0.7. The predictive performances of the two risk scores were similar, when comparing ROC curves (*P* = 0.294), and also when considering more advanced metrics such as IDI and NRI. However, the CHA_2_DS_2_-VASc (and its modified non-sex version, CHA_2_DS_2_-VA) was primarily designed to identify patients at low risk of stroke, considering OACs as the standard of care in patients with AF, and that OACs prescription should only be withhold in patients at very low risk of stroke (as identified by CHA_2_DS_2_-VASc/CHA_2_DS_2_-VA). These findings align with previous studies indicating the relatively weak predictive abilities of both scores, with some studies pointing towards a marginal statistical superiority of CHA_2_DS_2_-VA in more recent AF cohorts,^13–15^ albeit with unclear clinical significance.

One recent study that excluded AF patients aged ≥75 years and those with prior stroke, showed the ‘CHD-VA’ performed statistically better than the ‘CHD-VASc’ scores (AUCs: 0.651 vs. 0.639; *P* < 0.001) in predicting a composite of death, ischaemic stroke, and arterial TE, although the improvement was modest.^15^ However, the cohort included both new-onset and prevalent AF, complicating the interpretation of the results, as the clinical management of AF typically begins at the time of diagnosis. Furthermore, the exclusion of patients aged ≥75 and those with prior strokes likely resulted in a bias towards younger, healthier patients. Nevertheless, if one only focuses on the AUC, all risk scores based on clinical factors generally perform similarly,^19^ and the major limitations of focusing only on the AUC to compare risk prediction scores are well recognized.^20^

Additionally, studies demonstrated mixed results regarding the NRI and IDI for CHA_2_DS_2_-VA vs. CHA_2_DS_2_-VASc. For example, a Finnish cohort study found a marginally improved NRI and IDI with CHA_2_DS_2_-VA, whereas a UK cohort study reported no significant improvements, consistent with what we found in our analysis.^13,14^ Given their similar performances, the non-sex CHA_2_DS_2_-VASc (i.e. CHA_2_DS_2_-VA) may offer potentially some simplicity in initial decision-making for starting OAC in patients with AF,^21^ and (as the 2024 ESC Guidelines promote) would be inclusive of individuals who are ‘non-binary, transgender, or are undergoing sex hormone therapy.’^12^ On the other side, emphasis on female sex may increase awareness on the importance of equal treatment in patients with AF, thus increasing the uptake of OAC among women.^16^

This study elaborates on TE and stroke incidence in AF patients and demonstrates the performances of these two risk scores using contemporary global data. Recent epidemiological data suggest that sex-based differences in stroke incidence, and inequalities in stroke prevention, have diminished significantly;^7,10,11^ our data from a contemporary cohort of patients with recent diagnosis of AF seems to confirm these results, in a context of a broadly high uptake of OAC (>80%). Nonetheless, in the adjusted logistic regression analysis, we observed a slightly lower likelihood of receiving OACs in females, suggesting that such sex-based disparities may not be completely resolved, in accordance with other previous reports.^22^ These data may also reflect the fact that an additional point given to female sex in the CHA_2_DS_2_-VASc score may have led physicians to initiate OAC more frequently in females, thereby reducing inequities in care.^16^

We provided further insights into TE and stroke incidence by sex, categorized by CHA_2_DS_2_-VA and CHA_2_DS_2_-VASc scores. Our analysis shows that TE and stroke rates were similar between male and female patients, consistent with previous evidence. Additionally, TE and stroke rates for each point of the CHA_2_DS_2_-VA score were generally similar between sexes, though male patients with CHA_2_DS_2_-VA >3 had slightly higher (albeit non-significant) TE IR. In contrast, TE IR was slightly higher in males for each point of the CHA_2_DS_2_-VA score. This finding supports the idea of female sex as a risk modifier when other factors are present. Although CHA_2_DS_2_-VA perform similarly to CHA_2_DS_2_-VASc (with even slightly better performance in recent cohorts), whether sex should be treated as a risk modifier is still an important issue.^5^ Initially identifying low-risk patients is perhaps more important than identifying high-risk patients for clinician, since standard treatment should be OAC, unless patients are at (truly) low-risk.^21^

Our exploratory interaction analyses suggested that the association between female sex, the use of OAC, and the risk of stroke may be different across age spectrum. We observed lower odds of receiving OAC in younger females, with also some trends towards declining trends >70 years. These results, which should be interpreted with caution and regarded as exploratory, may suggest that younger male patients may be perceived at higher risk than females, thus being more likely to receive OAC when adjusting for other stroke risk factors; also, the lower odds of receiving OAC may explain the potential trends (albeit non-significant) towards higher risk of stroke in younger female patients. On the other side, the same trend towards higher risk in females at older ages (>70 years) may be explained by biological factors: previous studies indicate that younger females have lower stroke risks due to the protective effects of female hormones.^23,24^ Furthermore, epidemiological data reveal an association between reproductive lifespan and cardiovascular diseases.^25^ While further research is needed to explore the relationship between reproductive lifespan and cardiovascular outcomes in women with AF, our results provide interesting (though preliminary) insights on the sex-age interaction on both use of OAC and risk of thromboembolic events.

Taken together, and in accordance with previous studies, our results seem to suggest that the previously described sex-based differences in thromboembolic risk may no longer be evident in contemporary cohorts of patients with AF,^7,10,11^ likely being a result of the abated sex-based disparities in OAC use, and notwithstanding some potential residual differences as shown in our cohort. Whether these evolving trends can be ascribed to the introduction of NOAC, to the increasing awareness of female sex as a AF-related stroke risk factor (which has been bolstered by the inclusion of a specific point in the CHA_2_DS_2_-VASc score),^5^ or by a combination of both, remain an open question; whichever the reason, the higher uptake of OAC in females has highly contributed to mitigate the higher risk of stroke associated with female, and our exploratory sensitivity analysis on non-anticoagulated patients confirms this, by showing an increased risk of ischaemic stroke at 1-year in non-anticoagulated females compared with males. Indeed, over the years, the inclusion of the Sc (female sex) criterion into CHA_2_DS_2_-VASc could have helped increase the awareness of female sex as a risk modifier and mitigated the under-treatment of female AF patients with OAC.^6,7^ Whether the omission of the Sc criterion from CHA_2_DS_2_-VA would lead to less OAC in female AF patient over the next years remains to be seen.

### Strengths and limitations

This study is based on a global, contemporary, multicentre cohort of recently diagnosed AF patients with diverse geographical and ethnic representation. Our findings provide valuable epidemiological data for comparing the predictive performance of CHA_2_DS_2_-VA and CHA_2_DS_2_-VASc scores in stratifying stroke risk in AF patients. Notably, our cohort had a high uptake of OAC, thus broadly reflecting contemporary patterns of thromboembolic risk prevention for patients with AF.

However, this study has several limitations. First, as GLORIA-AF included only patients with a CHA_2_DS_2_-VASc ≥ 1, we had no patients at ‘low-risk’ (as identified by the CHA_2_DS_2_-VA score), and this limits our ability to focus on the specific subgroup of patients deemed at low-risk. To focus on the 1-year risk of thromboembolic events, we censored those events occurring after 1-year of follow-up, thus potentially reducing our power to detect some differences between groups. Also, while we adjusted our analysis for the factors included in the CHA_2_DS_2_-VA score (and additionally for use of OAC for the risk of major outcomes), we cannot exclude the contribution of other unaccounted confounders including physician bias on OAC prescription; finally our exploratory analysis on the sex-age interaction on the odds of receiving OAC and risk of thromboembolic events should be regarded as exploratory and may not have been adequately powered for detecting differences; therefore, these should be interpreted with caution.

## Conclusions

CHA_2_DS_2_-VASc and CHA_2_DS_2_-VA scores showed similar predictive performance for thromboembolic events. The role of female sex in the management and outcomes of patients with AF may differ according to age. Whether the omission of the female criterion from CHA_2_DS_2_-VA would lead to less OAC use in female AF patients over the next years remains to be seen.

## Supplementary Material

pvaf031_Supplementary_Data

## Data Availability

Data supporting this study by the data contributors Boehringer Ingelheim, and were made and are available through Vivli Inc. Access was provided after a proposal was approved by an independent review committee identified for this purpose and after receipt of a signed data sharing agreement.
