# Supplementary material for: Stroke risk stratifications according to CHA2DS2-VASc vs. CHA2DS2-VA in patients with atrial fibrillation: insights from the GLORIA-AF registry
Source: Eur Heart J Cardiovasc Pharmacother. 2025 Apr 28;11(5):433–40. doi: 10.1093/ehjcvp/pvaf031 (PMC12343043; doi:10.1093/ehjcvp/pvaf031)
Supplement: pvaf031_Supplementary_Data [file pvaf031_supplementary_data.zip › Revised Supplementary Materials 20250320.docx]

**Stroke risk stratifications according to CHA**_2_**DS**_2_**-VASc vs. CHA**_2_**DS**_2_**-VA in patients with Atrial Fibrillation: insights from the GLORIA-AF registry phase III**

Steven Ho Man Lam, PhD^1,2^*, Giulio Francesco Romiti, MD^1,3^*, Bernadette Corica, MD^1^, Tommaso Bucci, MD PhD^1,4^, Brian Olshansky, MD^5^,

Tze-Fan Chao, MD^6,7^, Menno V Huisman, MD^8^, Gregory Y. H. Lip, MD^1,9,10^;
on behalf of the GLORIA-AF Investigators

^1^Liverpool Centre for Cardiovascular Sciences at University of Liverpool, Liverpool John Moores University and Liverpool Heart & Chest Hospital, Liverpool, United Kingdom; ^2^Department of Medicine and Therapeutics, The Chinese University of Hong Kong; ^3^Department of Translational and Precision Medicine, Sapienza – University of Rome, Rome, Italy; ^4^Department of Clinical Internal, Anesthesiologic and Cardiovascular Sciences, Sapienza University of Rome, Rome, Italy; ^5^Division of Cardiology, Department of Medicine, University of Iowa, Iowa City, USA. ^6^Division of Cardiology, Department of Medicine, Taipei Veterans General Hospital, Taipei, Taiwan. ^7^Institute of Clinical Medicine, and Cardiovascular Research Center, National Yang Ming Chiao Tung University, Taipei, Taiwan. ^8^Department of Thrombosis and Hemostasis, Leiden University Medical Center, Leiden, the Netherlands; ^9^Danish Center for Health Services Research, Department of Clinical Medicine, Aalborg University, Aalborg, Denmark; ^10^Medical University of Bialystok, Bialystok, Poland.

*Joint first authors

**Correspondence:**

Prof. Gregory Y.H. Lip: E-mail: [gregory.lip@liverpool.ac.uk](mailto:gregory.lip@liverpool.ac.uk)

and

Prof. Tze-Fan Chao: E-mail: [eyckeyck@gmail.com](mailto:eyckeyck@gmail.com)

Supplementary Materials

**Table S1 – Incidence Rates and 95%CI and Incidence Rate Ratios (IRR) and 95%CI for Thromboembolism and Ischemic Stroke, according to scores and sex**

|  | | | | **CHA2DS2-VASc** | | | | | **CHA2DS2-VA** | | | |
| --- | --- | --- | --- | --- | --- | --- | --- | --- | --- | --- | --- | --- |
| **Score** | **Overall IR [95%CI]** | | **Females IR [95%CI]** | **Males IR [95%CI]** | | **Females vs. Males IRR [95%CI]** | **Overall IR [95%CI]** | | **Females IR [95%CI]** | **Males IR [95%CI]** | | **Females vs. Males  IRR [95%CI]** |
| **Thromboembolism** | |  | | |  | | |  | | |  | |
| 0 | - | | - | - | | - | 0.62 [0.13-1.81] | | 0.62 [0.13-1.81] | - | | - |
| 1 | 0.80 [0.51-1.18] | | 0.62 [0.13-1.81] | 0.83 [0.51-1.27] | | 0.75 [0.22-2.51] | 0.90 [0.63-1.25] | | 1.03 [0.56-1.72] | 0.83 [0.51-1.27] | | 1.24 [0.63-2.44] |
| 2 | 1.27 [0.96-1.65] | | 1.03 [0.56-1.72] | 1.38 [0.99-1.86] | | 0.75 [0.41-1.37] | 1.27 [0.99-1.61] | | 1.13 [0.75-1.65] | 1.38 [0.99-1.86] | | 0.82 [0.51-1.34] |
| 3 | 1.27 [0.98-1.61] | | 1.13 [0.75-1.65] | 1.38 [0.98-1.89] | | 0.82 [0.50-1.34] | 1.45 [1.14-1.81] | | 1.52 [1.07-2.08] | 1.38 [0.98-1.89] | | 1.10 [0.70-1.72] |
| 4 | 1.82 [1.43-2.28] | | 1.52 [1.07-2.08] | 2.28 [1.61-3.15] | | 0.66 [0.42-1.04] | 2.54 [2.00-3.17] | | 2.83 [2.02-3.86] | 2.28 [1.61-3.15] | | 1.24 [0.79-1.94] |
| 5 | 3.31 [2.61-4.15] | | 2.83 [2.02-3.86] | 4.11 [2.86-5.71] | | 0.69 [0.44-1.09] | 3.74 [2.86-4.82] | | 3.33 [2.16-4.92] | 4.11 [2.86-5.71] | | 0.81 [0.49-1.36] |
| ≥6 | 4.1 [3.13-5.27] | | 3.90 [2.80-5.29] | 4.59 [2.76-7.17] | | 0.85 [0.49-1.46] | 4.90 [3.41-6.81] | | 5.32 [3.04-8.65] | 4.59 [2.76-7.17] | | 1.16 [0.60-2.25] |
| **Ischemic Stroke** | |  | | |  | | |  | | |  | |
| 0 | - | | - | - | | - | 0.62 [0.13-1.81] | | 0.62 [0.13-1.81] | - | | - |
| 1 | 0.46 [0.25-0.78] | | 0.62 [0.13-1.81] | 0.43 [0.22-0.78] | | 1.43 [0.40-5.13] | 0.46 [0.27-0.73] | | 0.51 [0.21-1.06] | 0.43 [0.22-0.78] | | 1.18 [0.46-3.05] |
| 2 | 0.66 [0.44-0.94] | | 0.51 [0.21-1.06] | 0.72 [0.45-1.09] | | 0.71 [0.30-1.67] | 0.62 [0.43-0.87] | | 0.50 [0.26-0.88] | 0.72 [0.45-1.09] | | 0.70 [0.35-1.41] |
| 3 | 0.59 [0.40-0.84] | | 0.50 [0.26-0.88] | 0.67 [0.40-1.05] | | 0.75 [0.36-1.54] | 0.75 [0.54-1.02] | | 0.84 [0.52-1.28] | 0.67 [0.40-1.05] | | 1.24 [0.67-2.31] |
| 4 | 0.80 [0.55-1.12] | | 0.84 [0.52-1.28] | 0.74 [0.38-1.28] | | 1.14 [0.56-2.31] | 1.08 [0.75-1.52] | | 1.48 [0.92-2.27] | 0.74 [0.38-1.28] | | 2.02 [0.99-4.10] |
| 5 | 1.80 [1.29-2.45] | | 1.48 [0.92-2.27] | 2.33 [1.42-3.60] | | 0.64 [0.34-1.17] | 2.17 [1.51-3.02] | | 1.99 [1.11-3.28] | 2.33 [1.42-3.60] | | 0.85 [0.44-1.67] |
| ≥6 | 2.44 [1.71-3.38] | | 2.37 [1.53-3.49] | 2.64 [1.32-4.72] | | 0.90 [0.44-1.82] | 2.92 [1.81-4.46] | | 3.30 [1.58-6.08] | 2.64 [1.32-4.72] | | 1.25 [0.53-2.95] |

**Legend:** BMI= Body Mass Index; CAD= Coronary Artery Disease; COPD= Chronic Obstructive Pulmonary Disease; EHRA= European Heart Rhythm Association; IQR= Interquartile Range; PAD= Peripheral Artery Disease; SD= Standard Deviation; TIA= Transient Ischemic Attack.

**Table S2 – Incidence Rates and 95%CI and Incidence Rate Ratios (IRR) and 95%CI for Thromboembolism and Ischemic Stroke, according to scores and sex, in non-anticoagulated patients**

|  | | | **CHA2DS2-VASc** | | | **CHA2DS2-VA** | | |
| --- | --- | --- | --- | --- | --- | --- | --- | --- |
| **Score** | **Overall IR [95%CI]** | **Females IR [95%CI]** | **Males IR [95%CI]** | **Females vs. Males IRR [95%CI]** | **Overall IR [95%CI]** | **Females IR [95%CI]** | **Males IR [95%CI]** | **Females vs. Males  IRR [95%CI]** |
| **Thromboembolism** | | | | | | | | |
| 0 | - | - | - | - | 0.82 [0.10-2.96] | 0.82 [0.10-2.96] | - | - |
| 1 | 0.99 [0.45-1.87] | 0.82 [0.10-2.96] | 1.05 [0.42-2.16] | 0.78 [0.16-3.76] | 1.48 [0.81-2.48] | 2.51 [1.01-5.17] | 1.05 [0.42-2.16] | 2.39 [0.84-6.82] |
| 2 | 2.25 [1.31-3.61] | 2.51 [1.01-5.17] | 2.10 [1.01-3.87] | 1.19 [0.45-3.14] | 1.69 [0.93-2.84] | 1.14 [0.31-2.91] | 2.10 [1.01-3.87] | 0.54 [0.17-1.72] |
| 3 | 1.83 [0.97-3.12] | 1.14 [0.31-2.91] | 2.50 [1.14-4.75] | 0.45 [0.14-1.48] | 2.98 [1.85-4.56] | 3.48 [1.80-6.08] | 2.50 [1.14-4.75] | 1.39 [0.59-3.30] |
| 4 | 3.58 [2.21-5.47] | 3.48 [1.80-6.08] | 3.71 [1.70-7.05] | 0.94 [0.40-2.23] | 4.30 [2.59-6.71] | 5.01 [2.40-9.21] | 3.71 [1.70-7.05] | 1.35 [0.55-3.32] |
| 5 | 4.44 [2.48-7.32] | 5.01 [2.40-9.21] | 3.62 [1.17-8.44] | 1.38 [0.47-4.05] | 5.41 [2.96-9.07] | 7.45 [3.41-14.15] | 3.62 [1.17-8.44] | 2.06 [0.69-6.15] |
| ≥6 | 5.87 [3.29-9.68] | 7.10 [3.67-12.41] | 3.47 [0.71-10.13] | 2.05 [0.58-7.26] | 4.45 [1.63-9.69] | 6.23 [1.28-18.19] | 3.47 [0.71-10.13] | 1.80 [0.36-8.90] |
| **Ischemic Stroke** | | | | | | | | |
| 0 | - | - | - | - | 0.82 [0.10-2.96] | 0.82 [0.10-2.96] | - | - |
| 1 | 0.66 [0.24-1.43] | 0.82 [0.10-2.96] | 0.60 [0.16-1.53] | 1.37 [0.25-7.48] | 0.74 [0.30-1.52] | 1.07 [0.22-3.12] | 0.60 [0.16-1.53] | 1.79 [0.40-7.98] |
| 2 | 1.05 [0.46-2.08] | 1.07 [0.22-3.12] | 1.05 [0.34-2.44] | 1.02 [0.24-4.26] | 0.84 [0.34-1.74] | 0.57 [0.07-2.05] | 1.05 [0.34-2.44] | 0.54 [0.10-2.79] |
| 3 | 0.56 [0.15-1.43] | 0.57 [0.07-2.05] | 0.55 [0.07-2.00] | 1.02 [0.14-7.27] | 1.41 [0.68-2.60] | 2.31 [1.00-4.56] | 0.55 [0.07-2.00] | 4.18 [0.89-19.68] |
| 4 | 2.03 [1.05-3.55] | 2.31 [1.00-4.56] | 1.63 [0.45-4.18] | 1.41 [0.43-4.70] | 2.47 [1.23-4.42] | 3.49 [1.40-7.19] | 1.63 [0.45-4.18] | 2.13 [0.62-7.29] |
| 5 | 3.24 [1.62-5.80] | 3.49 [1.40-7.19] | 2.89 [0.79-7.39] | 1.21 [0.35-4.13] | 3.45 [1.58-6.56] | 4.10 [1.33-9.56] | 2.89 [0.79-7.39] | 1.42 [0.38-5.28] |
| ≥6 | 3.49 [1.60-6.63] | 4.11 [1.65-8.47] | 2.29 [0.28-8.27] | 1.80 [0.37-8.65] | 2.95 [0.80-7.55] | 4.15 [0.50-14.99] | 2.29 [0.28-8.27] | 1.81 [0.26-12.87] |

**Legend:** BMI= Body Mass Index; CAD= Coronary Artery Disease; COPD= Chronic Obstructive Pulmonary Disease; EHRA= European Heart Rhythm Association; IQR= Interquartile Range; PAD= Peripheral Artery Disease; SD= Standard Deviation; TIA= Transient Ischemic Attack.

; SD= Standard Deviation; TIA= Transient Ischemic Attack.

**Table S3 – Integrated discrimination improvement index (IDI) and continuous net reclassification index (NRI) at 1 year for CHA2DS2-VA vs. CHA_2_DS_2_-VASc, on thromboembolism and ischemic stroke.**

| **Metric** | **Thromboembolism** | **Ischemic Stroke** |
| --- | --- | --- |
| IDI [95%CI] | 0.001 [0.000; 0.001], p=0.233 | 0.001 [0.000; 0.001], p=0.824 |
| NRI [95%CI] | -0.007 [-0.060; 0.045], p=0.771 | -0.035 [-0.104; 0.035], p=0.439 |

**Legend:** CI= Confidence Interval; IDI= Integrated Discrimination Improvement index; NRI= Net Reclassification Index

**Table S4 – Integrated discrimination improvement index (IDI) and continuous net reclassification index (NRI) at 1 year for CHA2DS2-VA vs. CHA_2_DS_2_-VASc, on thromboembolism and ischemic stroke, in non-anticoagulated patients.**

| **Metric** | **Thromboembolism** | **Ischemic Stroke** |
| --- | --- | --- |
| IDI [95%CI] | -0.001 [-0.003; 0.001], p=0.346 | -0.001 [-0.004; 0.000], p=0.093 |
| NRI [95%CI] | -0.073 [-0.171; 0.034], p=0.186 | -0.137 [-0.302; 0.005], p=0.060 |

**Legend:** CI= Confidence Interval; IDI= Integrated Discrimination Improvement index; NRI= Net Reclassification Index

**Figure S1 – Antithrombotic Treatment at baseline, stratified by score**


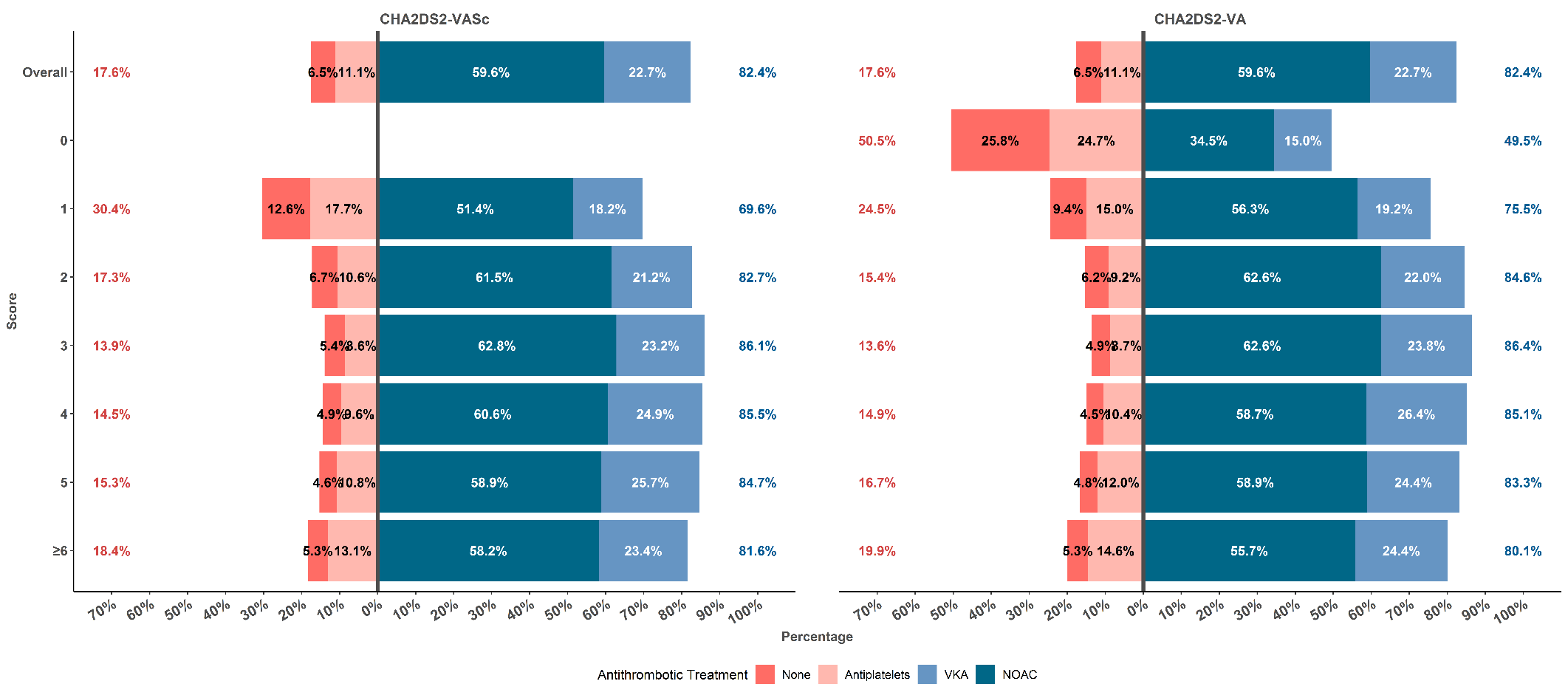


**Legend:** NOAC= Non-vitamin K antagonist oral anticoagulant; VKA= Vitamin K Antagonist

**Figure S2 – Incidence Rates and 95% Confidence Intervals for Thromboembolism and Ischemic Stroke, according to sexes and scores**


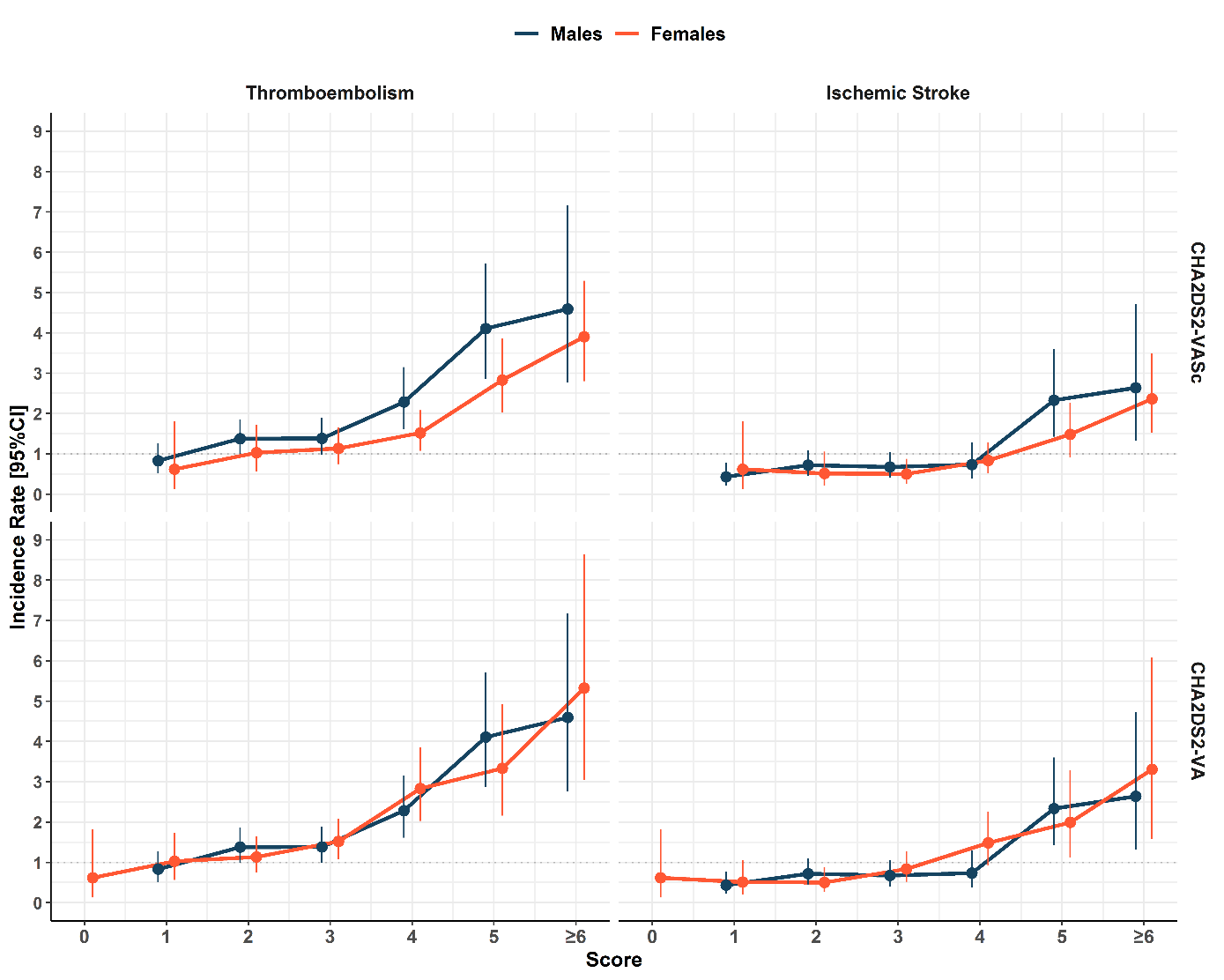


**Legend:** CI= Confidence Interval

**Figure S3 – Hazard Ratio and 95% Confidence Intervals for Thromboembolism (Left Panel) and Ischemic Stroke (Right Panel) in Females vs. Males patients, according to age**


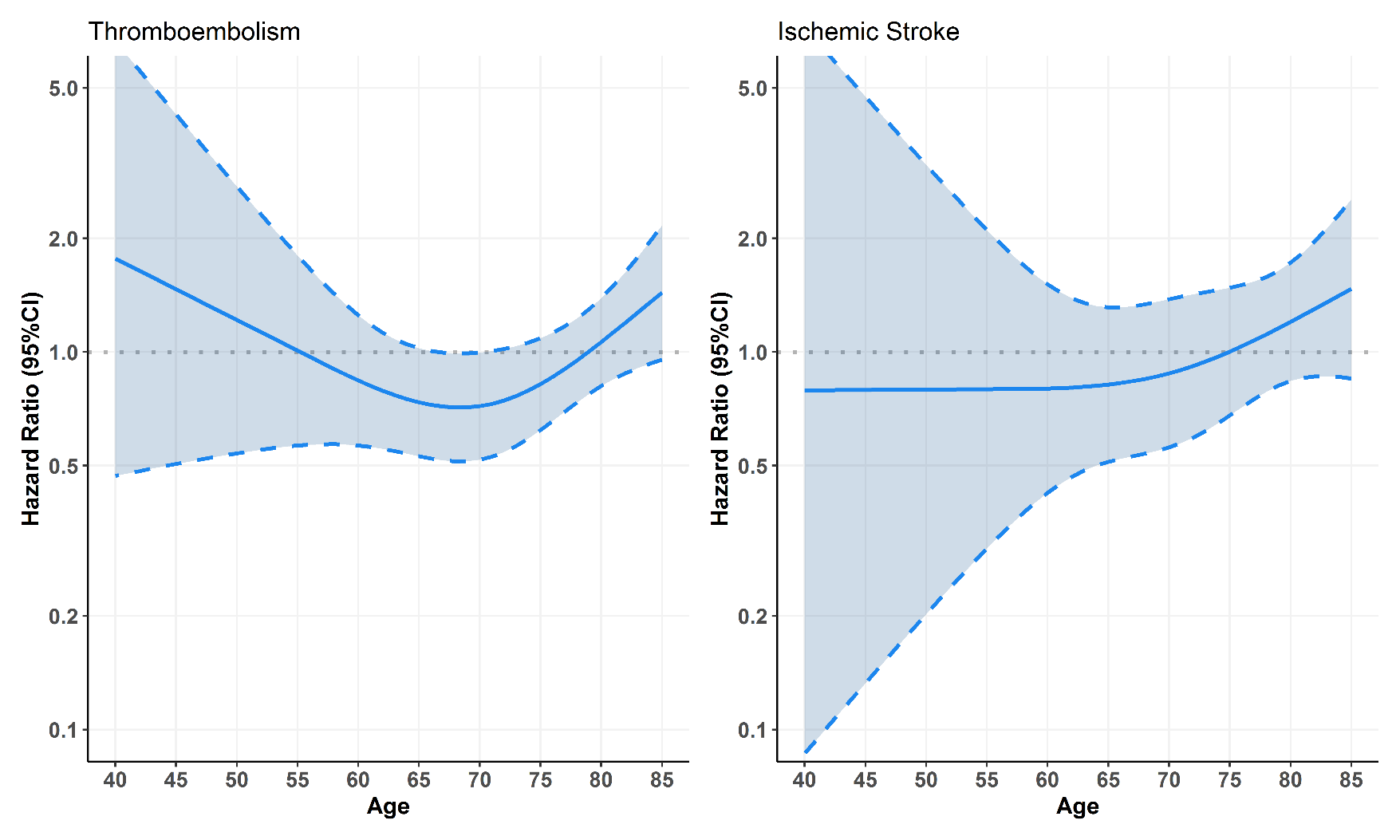


**Legend:**CI= Confidence Intervals

**Figure S4 – ROC Curves for Thromboembolism and Ischemic Stroke, according to scores, in non-anticoagulated patients**


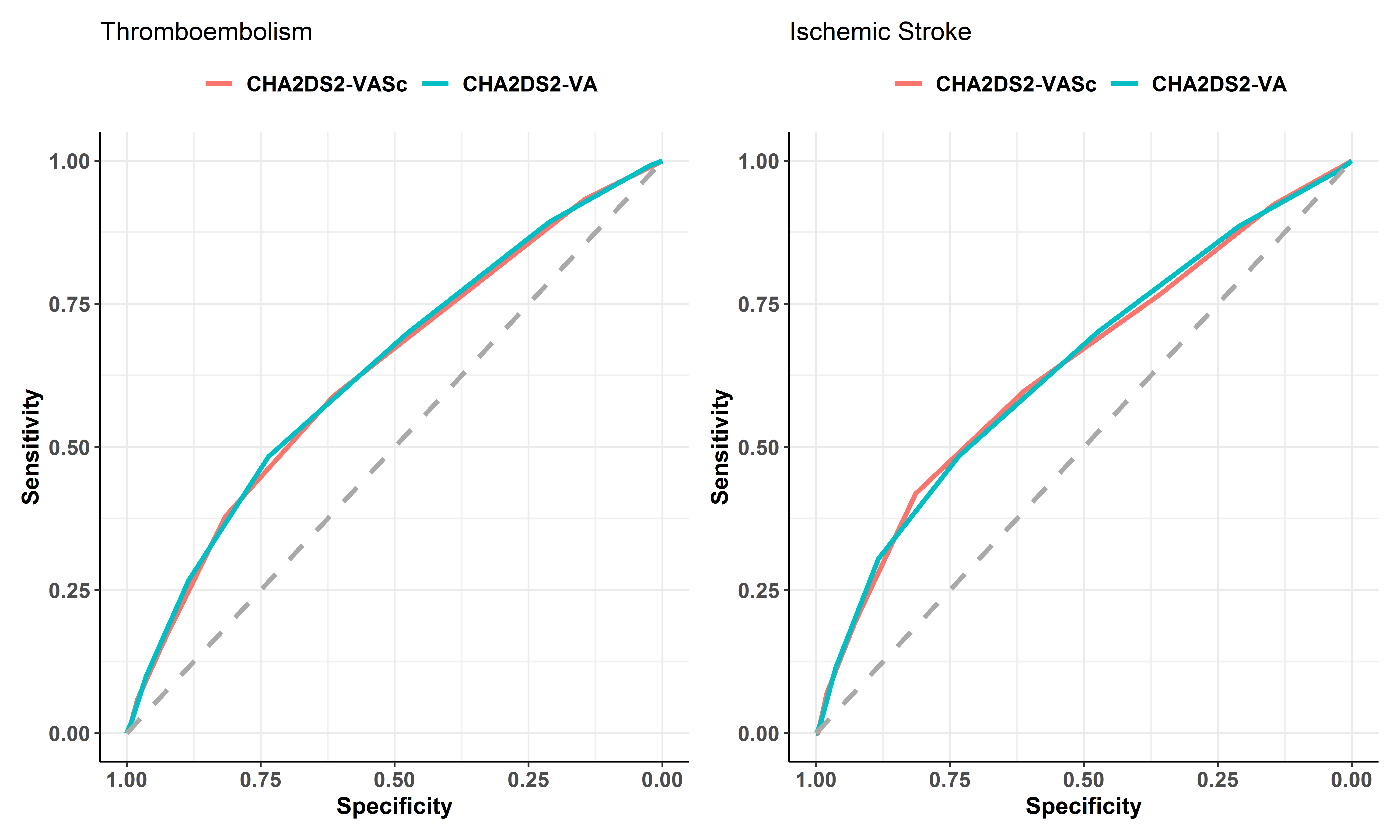


**Figure S5 – Hazard Ratio and 95% Confidence Intervals for Thromboembolism (Left Panel) and Ischemic Stroke (Right Panel) in Females vs. Males patients, according to age, in non-anticoagulated patients**


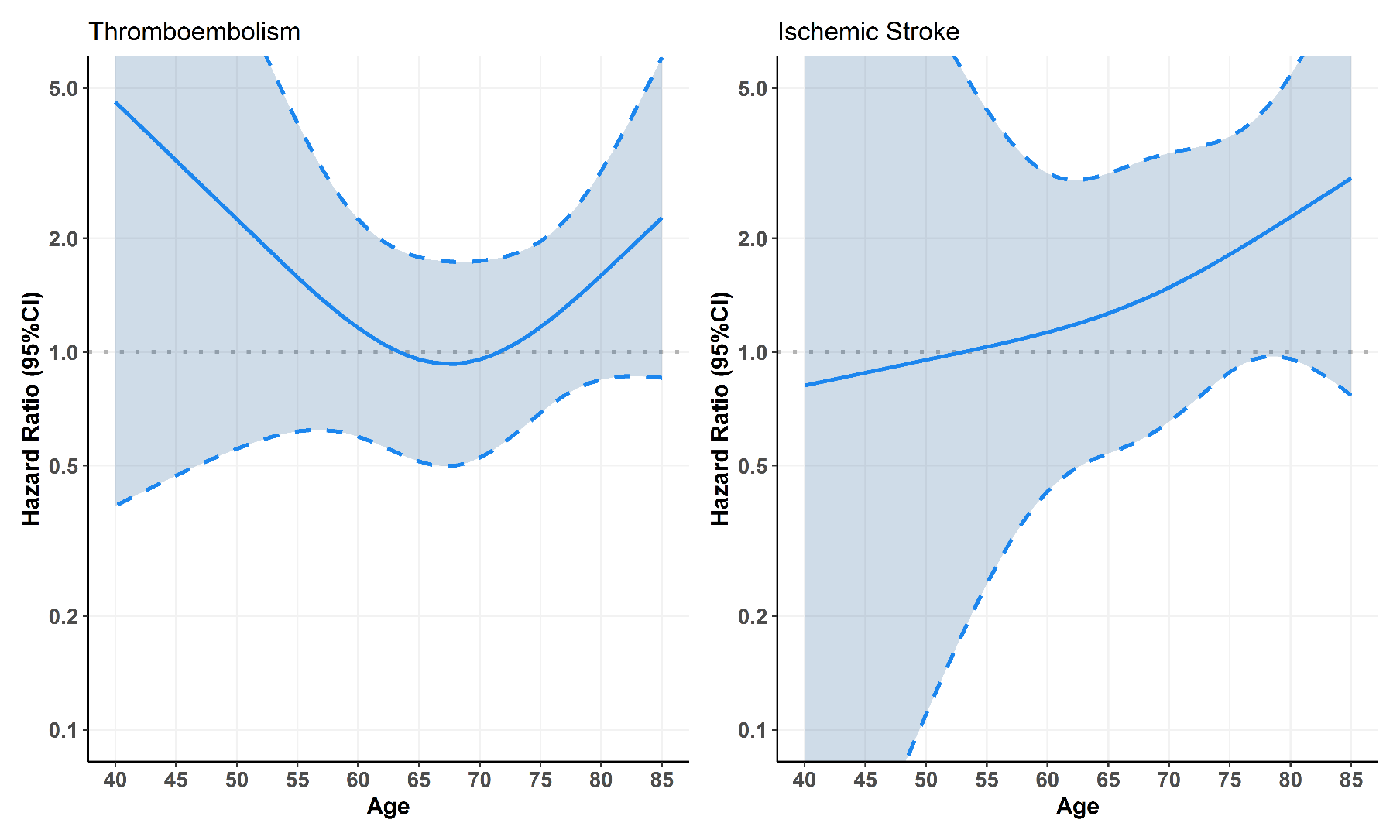


**Legend:** CI= Confidence Intervals
